# Supplementary material for: Neolithic and medieval virus genomes reveal complex evolution of hepatitis B
Source: eLife. 2018 May 10;7:e36666. doi: 10.7554/eLife.36666 (PMC6008052; doi:10.7554/eLife.36666)
Supplement: Supplementary file 1. — Since monkey HBV strains are not classified into genotypes the column is left blank. [file elife-36666-supp1.docx]

**Supplementary File 1.** Accession numbers for the reference genomes used in the first alignment step to catch HBV diversity in the sample. Since monkey HBV strains are not classified into genotypes the column is left blank.

| Accession | Genotype | Host |
| --- | --- | --- |
| X51970 | A | Human |
| AB073846.1 | B | Human |
| M12906 | C | Human |
| M32138 | D | Human |
| AB032431 | E | Human |
| AB036910 | F | Human |
| AB064310 | G | Human |
| AY090454 | H | Human |
| AB032433 |  | Chimpanzee |
| AF222323 |  | Chimpanzee |
| AJ131567 |  | Gorilla |
| AY330911.1 |  | Chimpanzee |
| AJ131571.1 |  | Gibbon |
| U46935.1 |  | Gibbon |
| FM209516.1 |  | Gibbon |
| AF193863.1 |  | Orangutan |
