# Supplementary material for: Neolithic and medieval virus genomes reveal complex evolution of hepatitis B
Source: eLife. 2018 May 10;7:e36666. doi: 10.7554/eLife.36666 (PMC6008052; doi:10.7554/eLife.36666)
Supplement: Supplementary file 2. [file elife-36666-supp2.docx]

**Supplementary File 2.** Number of reads mapping against the references shown in Table S1 before and after duplicate removal.

| Sample | Number of reads mapping | After duplicate removal |
| --- | --- | --- |
| Karsdorf | 12182 | 10718 |
| Sorsum | 5949 | 4299 |
| Petersberg | 2729 | 2125 |
