# Supplementary material for: Neolithic and medieval virus genomes reveal complex evolution of hepatitis B
Source: eLife. 2018 May 10;7:e36666. doi: 10.7554/eLife.36666 (PMC6008052; doi:10.7554/eLife.36666)
Supplement: Supplementary file 3. [file elife-36666-supp3.docx]

**Supplementary File 3.** Number of contigs and combined contig length of the *de novo* assembly for choosen K-values.

| Sample | Choosen K-value | Number of contigs | Combined contig length |
| --- | --- | --- | --- |
| Karsdorf | 17 | 3 | 3207 |
| Sorsum | 41 | 1 | 3276 |
| Petersberg | 17 | 2 | 2692 |
