# Supplementary material for: Neolithic and medieval virus genomes reveal complex evolution of hepatitis B
Source: eLife. 2018 May 10;7:e36666. doi: 10.7554/eLife.36666 (PMC6008052; doi:10.7554/eLife.36666)
Supplement: Supplementary file 4. [file elife-36666-supp4.docx]

**Supplementary File 4.** Final consensus length after retrieving gap information from the multiple sequence alignment with Geneious.

| Sample | Consensus length |
| --- | --- |
| Karsdorf | 3183 |
| Sorsum | 3182 |
| Petersberg | 3161 |
