# Supplementary material for: Neolithic and medieval virus genomes reveal complex evolution of hepatitis B
Source: eLife. 2018 May 10;7:e36666. doi: 10.7554/eLife.36666 (PMC6008052; doi:10.7554/eLife.36666)
Supplement: Supplementary file 5. [file elife-36666-supp5.docx]

**Supplementary File 5.**

| Sample | Number of mapped reads | Number of mapped reads (after DeDup) | Coverage >=1x |
| --- | --- | --- | --- |
| Karsdorf | 131860289 | 122568310 | 70,2% |
| Sorsum | 25300485 | 9856001 | 18.8% |
| Petersberg | 143954577 | 105476677 | 77,4% |
