# Supplementary material for: Neolithic and medieval virus genomes reveal complex evolution of hepatitis B
Source: eLife. 2018 May 10;7:e36666. doi: 10.7554/eLife.36666 (PMC6008052; doi:10.7554/eLife.36666)
Supplement: Supplementary file 6. — Shown are mean coverage, mean coverage for the covered region, genome length, number of missing bases and covered bases [file elife-36666-supp6.docx]

**Supplementary File 6.** Basic statistics for the mapping against the references shown in table S1. Shown are mean coverage, mean coverage for the covered region, genome length, number of missing bases and covered bases

| **Karsdorf** |  |  |  |  |  |
| --- | --- | --- | --- | --- | --- |
| Reference | Mean coverage | Mean coverage cov. | Genome length | Bases missing | Covered Bases |
| A | 4,12139 | 10,0492 | 3221 | 1900 | 1321 |
| gibbon_orang_1_restored | 5,96826 | 12,4941 | 3182 | 1662 | 1520 |
| B | 3,46345 | 9,69104 | 3215 | 2066 | 1149 |
| C | 20,6267 | 44,3876 | 3215 | 1721 | 1494 |
| AY330911.1_chimp_gorilla | 16,0003 | 22,3205 | 3182 | 901 | 2281 |
| D | 24,7942 | 42,0773 | 3182 | 1307 | 1875 |
| U46935.1_gibbon_orang | 12,8231 | 24,1581 | 3182 | 1493 | 1689 |
| E | 16,0311 | 24,3922 | 3212 | 1101 | 2111 |
| F | 1,06065 | 4,2203 | 3215 | 2407 | 808 |
| AF193863.1_gibbon_orang | 12,6266 | 26,4677 | 3182 | 1664 | 1518 |
| G | 24,1961 | 41,7139 | 3248 | 1364 | 1884 |
| chimp_gorilla_3_resotred | 13,1691 | 17,8239 | 3182 | 831 | 2351 |
| H | 2,37574 | 11,0856 | 3215 | 2526 | 689 |
| AB032433_chimp_gorilla | 18,5654 | 26,5386 | 3182 | 956 | 2226 |
| FM209516.1_gibbon_orang | 5,4428 | 13,436 | 3182 | 1893 | 1289 |
| AF222323_chimp_gorilla | 16,456 | 23,7474 | 3182 | 977 | 2205 |

| **Sorsum** |  |  |  |  |  |
| --- | --- | --- | --- | --- | --- |
| Reference | Mean coverage | Mean coverage cov. | Genome length | Bases missing | Covered Bases |
| A | 2,23812 | 5,54965 | 3221 | 1922 | 1299 |
| gibbon_orang_1_restored | 3,08674 | 6,65898 | 3182 | 1707 | 1475 |
| B | 1,87278 | 4,49664 | 3215 | 1876 | 1339 |
| C | 4,70638 | 12,1048 | 3215 | 1965 | 1250 |
| AY330911.1_chimp_gorilla | 8,39189 | 13,4321 | 3182 | 1194 | 1988 |
| D | 6,54808 | 12,9095 | 3182 | 1568 | 1614 |
| U46935.1_gibbon_orang | 4,60245 | 8,99018 | 3182 | 1553 | 1629 |
| E | 5,82067 | 10,5152 | 3212 | 1434 | 1778 |
| F | 1,59285 | 6,02471 | 3215 | 2365 | 850 |
| AF193863.1_gibbon_orang | 2,47046 | 6,00535 | 3182 | 1873 | 1309 |
| G | 4,47691 | 7,56162 | 3248 | 1325 | 1923 |
| chimp_gorilla_3_resotred | 6,19485 | 10,3638 | 3182 | 1280 | 1902 |
| H | 1,67683 | 8,25574 | 3215 | 2562 | 653 |
| AB032433_chimp_gorilla | 8,78536 | 14,4695 | 3182 | 1250 | 1932 |
| FM209516.1_gibbon_orang | 5,39126 | 12,7168 | 3182 | 1833 | 1349 |
| AF222323_chimp_gorilla | 13,336 | 20,0354 | 3182 | 1064 | 2118 |

| **Petersberg** |  |  |  |  |  |
| --- | --- | --- | --- | --- | --- |
| Reference | Mean coverage | Mean coverage cov. | Genome length | Bases missing | Covered Bases |
| A | 1,52313 | 4,92076 | 3221 | 2224 | 997 |
| gibbon_orang_1_restored | 0,434632 | 3,34867 | 3182 | 2769 | 413 |
| B | 0,970451 | 3,97959 | 3215 | 2431 | 784 |
| C | 1,90513 | 6,30144 | 3215 | 2243 | 972 |
| AY330911.1_chimp_gorilla | 0,69296 | 2,54032 | 3182 | 2314 | 868 |
| D | 22,9139 | 25,3255 | 3182 | 303 | 2879 |
| U46935.1_gibbon_orang | 0,2467 | 2,13315 | 3182 | 2814 | 368 |
| E | 8,81071 | 18,2228 | 3212 | 1659 | 1553 |
| F | 0,167341 | 1,92143 | 3215 | 2935 | 280 |
| AF193863.1_gibbon_orang | 0,335324 | 2,02467 | 3182 | 2655 | 527 |
| G | 1,12192 | 6,11409 | 3248 | 2652 | 596 |
| chimp_gorilla_3_resotred | 0,226587 | 1,79353 | 3182 | 2780 | 402 |
| H | 0,298289 | 2,42172 | 3215 | 2819 | 396 |
| AB032433_chimp_gorilla | 0,461031 | 2,10776 | 3182 | 2486 | 696 |
| FM209516.1_gibbon_orang | 0,268699 | 1,36581 | 3182 | 2556 | 626 |
| AF222323_chimp_gorilla | 0,804525 | 3,44549 | 3182 | 2439 | 743 |
